# Marfan Syndrome Caused by Disruption of the *FBN1* Gene due to A Reciprocal Chromosome Translocation

**DOI:** 10.3390/genes12111836

**Published:** 2021-11-21

**Authors:** Anna Clara Schnause, Katalin Komlosi, Barbara Herr, Jürgen Neesen, Paul Dremsek, Thomas Schwarz, Andreas Tzschach, Sabine Jägle, Ekkehart Lausch, Judith Fischer, Birgitta Gläser

**Affiliations:** 1 Institute of Human Genetics, Medical Center-University of Freiburg, Faculty of Medicine, University of Freiburg, 79106 Freiburg, Germany; anna.schnause@uniklinik-freiburg.de (A.C.S.); katalin.komlosi@uniklinik-freiburg.de (K.K.); barbara.herr@uniklinik-freiburg.de (B.H.); andreas.tzschach@uniklinik-freiburg.de (A.T.); sabine.jaegle@uniklinik-freiburg.de (S.J.); judith.fischer@uniklinik-freiburg.de (J.F.); 2 Institute of Medical Genetics, Center for Pathobiochemistry and Genetics, University of Vienna, 1090 Vienna, Austria; juergen.neesen@meduniwien.ac.at (J.N.); paul.dremsek@meduniwien.ac.at (P.D.); thomas.schwarz@meduniwien.ac.at (T.S.); 3 Department of General Pediatrics, Adolescent Medicine and Neonatology, Faculty of Medicine, University of Freiburg, 79106 Freiburg, Germany; ekkehart.lausch@uniklinik-freiburg.de

**Keywords:** *FBN1*, Marfan syndrome, apparently balanced chromosomal rearrangements (ABCR), optical genome mapping (OGM), gene disruption

## Abstract

Marfan syndrome (MFS) is a hereditary connective tissue disease caused by heterozygous mutations in the fibrillin-1 gene (*FBN1*) located on chromosome 15q21.1. A complex chromosomal rearrangement leading to MFS has only been reported in one case so far. We report on a mother and daughter with marfanoid habitus and no pathogenic variant in the *FBN1* gene after next generation sequencing (NGS) analysis, both showing a cytogenetically reciprocal balanced translocation between chromosomes 2 and 15. By means of fluorescence in situ hybridization of Bacterial artificial chromosome (BAC) clones from the breakpoint area on chromosome 15 the breakpoint was narrowed down to a region of approximately 110 kb in *FBN1*. With the help of optical genome mapping (OGM), the translocation breakpoints were further refined on chromosomes 2 and 15. Sequencing of the regions affected by the translocation identified the breakpoint of chromosome 2 as well as the breakpoint of chromosome 15 in the *FBN1* gene leading to its disruption. To our knowledge, this is the first report of patients with typical clinical features of MFS showing a cytogenetically reciprocal translocation involving the *FBN1* gene. Our case highlights the importance of structural genome variants as an underlying cause of monogenic diseases and the useful clinical application of OGM in the elucidation of structural variants.

## 1. Introduction

Marfan syndrome (MFS) as an autosomal-dominant disorder is the most common hereditary connective tissue disease, with a defect in the synthesis of microfibrils caused by heterozygous pathogenic variants in the fibrillin-1 gene (*FBN1*) located on chromosome 15q21.1 [1]. Fibrillin is the major constitutive element of extracellular microfibrils and has widespread distribution in both elastic and nonelastic connective tissue throughout the human body. Pathogenic *FBN1* variants lead to a disruption in the incorporation of the microfibrils into the extracellular matrix. This can affect different organ systems such as the cardiovascular system, eyes, and skeleton [2]. The diagnostic assessment of Marfan syndrome is complex due to its variability in age of onset, tissue distribution, severity of clinical features, and a variety of differential diagnosis. The clinical diagnosis of MFS is based on the 2010 revision of the Ghent nosology criteria by fulfilling at least two of the following four criteria: *FBN1* mutation, lens dislocation, aorta root widening or aortic root dissection, and clinical score. The nosology emphasizes the cardinal clinical features of aortic root aneurysm/dissection and ectopia lentis, and a more prominent role is attributed to molecular genetic testing of *FBN1*. Without family history, the presence of the two cardinal manifestations is sufficient. In absence of either one of these two, the presence of an *FBN1* mutation or a combination of systemic manifestations contributing to a systemic score ≥ 7 points is necessary [3].

So far, the Human Gene Mutation Database (HGMD® Professional version 2021.3, http://www.biobase-international.com/product/hgmd, access date 15 November 2021) has reported 2860 different disease-causing mutations, 2561 of them for MFS [4], across the entire *FBN1* gene with missense mutations being the most common [5]. Other single nucleotide variants (SNV) such as nonsense, splice site, or frameshift mutations as well as intragenic deletions have been reported [6,7]. Further, only one case of a patient carrying a complex chromosome rearrangement that resulted in the heterozygous deletion of *FBN1* has been described [8]. However, the diagnosis remains often challenging due to the high inter- and intrafamilial phenotype variability. Studies of genotype–phenotype associations have yielded few correlations, with the exception of neonatal Marfan syndrome, where previous studies have shown clustering of variants in exons 25–33, and ectopia lentis [9,10,11,12,13]. To the best of our knowledge, no patient with Marfan syndrome and a chromosome breakpoint in *FBN1* has been described so far.

## 2. Materials and Methods

### 2.1. Patients

This study was conducted according to the Declaration of Helsinki principles and approved by Ethics Committee of the University of Freiburg (21-1364). Peripheral blood samples of the affected mother, the affected daughter, the unaffected father, and the unaffected daughter were collected after written informed consent was obtained from the patients, and DNA was isolated according to standard procedures. 

### 2.2. Panel Diagnostics

For NGS panel diagnostics (including the genes *COL5A1, COL5A2, COL1A1, COL1A2, TNXB, TGFR1, SMAD3, TGFBR2, COL3A1, FBN1,* and *TGFBR2*) DNA sequences were enriched by a SureSelect Custom Kit (Agilent Technologies, Inc., Santa Clara, CA, USA). Resulting data were analyzed using an in-house bioinformatics pipeline and the commercial software SequencePilot (JSI medical systems, Ettenheim, Germany). Additionally, multiplex ligation-dependent probe amplification (MLPA) (P065-C1 and P066-C1, MRC Holland, Amsterdam, NL, USA) was performed for the *FBN1* gene.

### 2.3. Conventional Cytogenetic Analysis

**Figure 1:**
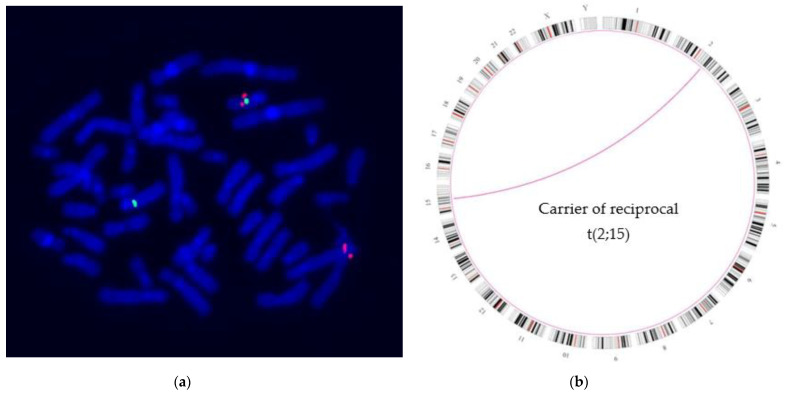
(**a**) Example of a FISH analysis with BAC RP11-498N3 (15q21.1 in red) located distally and BAC RP11-26I19 (15q15.3 in green) (Empire Genomics, Inc., New York, NY, USA) located proximal to the translocation breakpoint on chromosome 15, the derivative chromosome 15 shows the signal of BAC RP11-26I19 while the signal of BAC RP11-498N3 is on the derivative chromosome 2; (**b**) Optical genome mapping (OGM) shows the Circos plot view with the ideograms (G-banding, Black and gray: Giemsa positive. Red: Centromere) of the 24 chromosomes. The purple line points to the translocation observed between chromosomes 2 and 15.

Conventional chromosome analysis from peripheral blood lymphocytes was performed by replicaton banding by acridinorange (RBA-banding), and analyzed with Ikaros Karyotyping System V 5.0 (Metasystems Inc., Altussheim, Germany, Figure 1a) according to standard protocols with a resolution of ≈550 band level.

### 2.4. Microarray Based Molecular Cytogenomic Analysis

Genome-wide CNV detection was performed by microarray-analysis (CytoSure^®^ Constitutional v3 Array 180k, OGT ((© Oxford Gene Technology IP Limited, Begbroke, Oxford, UK) according to the manufacturer’s instructions. After hybridization, the array was scanned with the SureScan Microarray scanner (Agilent Technologies, Santa Clara, CA, USA) and analyzed using CytoSure™ interpret software v4.11 (© Oxford Gene Technology IP Limited, Begbroke, Oxford, UK ) based on the Genome Reference Consortium human genome GRCh37 (hg19).

### 2.5. Molecular Cytogenetic Analysis

Subsequent fluorescence in situ hybridizations (FISH) with human-derived DNA probes were performed according to standard protocols. Region-specific fluorescence-labeled BAC (Bacterial Artificial Chromosomes) clones (Empire Genomics, Inc., New York, NY, USA) were used for chromosomes 2q and 15q. Cell images were captured with the use of Isis Digital Imaging System V 5.0 (Metasystem Inc., Altussheim, Germany).

### 2.6. Optical Genome Mapping and Sequencing

For optical genome mapping, DNA was isolated from leucocytes using the SP Blood & Cell Culture DNA Isolation Kit (Bionano Genomics, Inc., San Diego, CA, USA), according to the manufacturer’s protocol “SP Frozen Human Blood DNA Isolation”. Thereafter, 750 ng of the DNA was labelled using the DLS DNA Labeling Kit (Bionano Genomics, Inc. San Diego, CA, USA) according to the manufacturer’s instructions. The DNA was applied onto a G1.2 flow cell and analyzed on a Saphyr instrument (Bionano Genomics). The data was analyzed using the software modules Tools (1.6.1), Solve (3.6.1_11162020), RefAligner (11643), Pipeline (11646) (Bionano Genomics, Figure 1b), and GRCh38 (hg38) as a reference. However, all positions are given in reference to hg19.

Amplification by PCR of the regions affected by the translocation was done using primers 5′-CCCTTTCCAATTGCAGTACCTCTTCAGT-3′ and 5′-ACCTCCTGAACACCTGCAATTTCCTAAG-3′ (yielding a product of 3267 bp in size), as well as 5′-AGCTGCATCATTCATTTGATATTTAGTTATATATAC-3′ and 5′-GTCTCATAAATAATTCCTCTACATGTTTTCTTTATC-3′ (yielding a product of 6724 bp in size). Both amplicons were sequenced using the SQK-LSK109 Kit (Oxford Nanopore Technologies, Oxford, UK), a FLO-MIN106 Flowcell (Oxford Nanopore Technologies), and a MinIon sequencer with fast basecalling (Oxford Nanopore Technologies).

## 3. Results

### 3.1. Clinical Report

The two patients, presenting the clinical picture of a connective tissue disease, were seen at the genetic counseling unit of our institute. Both patients showed marfanoid habitus, but with intrafamilial variability (Figures S1 and S2).

Patient 1 is a 40-year-old woman (Figure S1), who suffers from joint pain, congenital strabismus, and significant visual impairment due to myopia since childhood. Her pronounced foot deformity (hindfoot varus after correction, hallux valgus, malposition of the left toe D1) led to several operations. She had reduced exercise tolerance due to muscle weakness and muscle hypotrophy of the forearms and calves. Skeletal manifestations included arachnodactyly, joint laxity, and pectus carinatum deformity. She is 172 cm tall, has an arm span of 180 cm (arm span/height ratio: 1.047), and weighs 86 kg (BMI: 29.1)

Patient 2 is the older daughter of patient 1 (Figure S1), who fulfilled the Ghent criteria (aortic root widening and a clinical score of 8) for Marfan syndrome and showed congenital genua valga and pedes planovalgi. She had a dorsal repositioning spondylodesis due to her right convex lumbar scoliosis at the age of 15. Due to a 42 mm root aneurysm, valve-preserving aortic root replacement took place at the age of 19. She suffers from joint and back pain, joint instability, and susceptibility to hematomas. At the age of 19, she is 182 cm tall, has an arm span of 189 cm (arm span/height ratio: 1.038), and weighs 64 kg (BMI: 19.3).

The father of patient 1 (Figure S2), who was about 200 cm tall, and a paternal uncle, had skeletal abnormalities including abnormalities of the chest. Both died of sudden death in young adulthood. Cardiac death was suspected in the father of patient 1. The paternal grandfather of patient 1 had heart problems and died suddenly at the age of 40.

### 3.2. Cytogenetic, Cytogenomic, and Molecular Genetic Results

Based on the clinical findings, Marfan syndrome (MFS) was initially suspected in the daughter and the mother. Molecular genetic analyses revealed no pathogenic or likely pathogenic variants in the genes *COL5A1, COL5A2, COL1A1, COL1A2, TNXB, TGFR1, SMAD3, TGFBR2, COL3A1, FBN1,* and *TGFBR2* in the mother and the daughter.

During the routine work-up of the sample of the mother, however, karyotyping revealed a reciprocal translocation in the mother, and the same was confirmed in the daughter: 46,XX,t(2; 15)(q22; q21.1). The unaffected younger daughter (age 15 years) does not carry the reciprocal translocation. Routine clinical chromosomal microarray analysis (CMA) from blood lymphocytes revealed no disease-causing microdeletions or microduplications in either patient and no imbalance in the breakpoints.

In order to further elucidate the apparently balanced translocation and to determine the translocation breakpoints more precisely, fluorescence in situ hybridizations with different BAC probes from the translocation regions of chromosomes 2 and 15 were carried out in both patients. As the BAC probe RP11-154J22 (15q21.1) (g.48509039-48663778,hg19) hybridized on the normal chromosome 15 and on the derivative chromosome 15 and the BAC probe RP11-498N3 (15q21.1) (g.48772339-48869186, hg19) hybridized on the normal chromosome 15 and the derivative chromosome 2 (Figure 1a), the translocation breakpoint on chromosome 15q21.1 could be limited to the area of about 110 kb from g.48663778 proximal the 3′ end of *FBN1* to g.48772339 (GRCh37, hg19).

The *FBN1* gene starts at the 3′ end with g.48700503 and ends with g.48938046 in region 5′ (GRCh37, hg19). According to the FISH results, there was the possibility that the translocation breakpoint lies either within the *FBN1*, resulting in a loss of function of the *FBN1* gene, or it lies outside the *FBN1* gene not far from the 3′ region. This could have an impact on the regulatory elements of the *FBN1* gene. In addition to conventional cytogenetics, OGM confirmed the assumption of a balanced translocation involving the chromosomes 2 and 15 with a resolution of approximately 11 kb (Figure 1b). The breakpoints are located between g.139888511 and g.139898832 of chromosome 2 and between g.48716726 and g.48724425 of chromosome 15.

**Figure 2:**
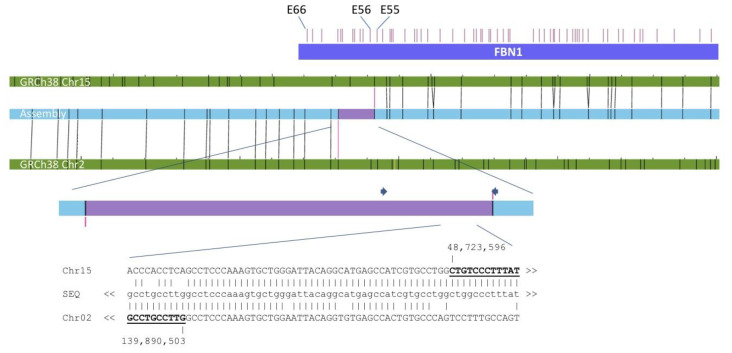
Optical map depicting the breakpoint within the *FBN1* gene, leading to its disruption. The region was amplified by PCR (primer depicted as grey arrows) and sequenced, further specifying the breakpoint being located within intron 55 of the *FBN1* gene. The alignment of the sequenced amplicon (SEQ) to the corresponding regions of chromosomes 2 and 15 revealed a 45 base pair region difficult to map to either of the chromosomes due to high sequence homology.

To further narrow down the breakpoints identified by OGM, long-range amplicons spanning the breakpoints were created and sequenced using Nanopore technology (Figure 2). By that, we were able to further narrow down the breakpoint located on chromosome 2 to chr2:139890503 and chr15:48723596 with two base pairs in between, mappable to both chromosomes. The other breakpoint located on chromosome 15 was specified at chr2:139896960 and chr15:48721471 with a 45 base pair region in between that was mappable to either of the chromosomes due to high sequence homology. This data suggests that there are minor losses of approximately 6.4 kb and 2.2 kb on chromosome 2 and 15, respectively.

The breakpoint of chromosome 15 lies within intron 55 of the *FBN1* gene. Consequently, the disruption of *FBN1* is most likely the cause of the marfanoid phenotype seen in the mother and her daughter.

## 4. Discussion

In general, apparently balanced chromosomal rearrangements (ABCR) associated with an abnormal phenotype are rare events and are usually challenging for genetic counselling, since molecular characterization of breakpoints is not yet performed in routine diagnostics [14].

Usually, ABCR are not associated with abnormal phenotypes and thus lead to an interpretational and counseling dilemma in cases with conspicuous phenotypes [15]. However, on the chromosomal or molecular level, patients with a reciprocal chromosomal rearrangement often reveal imbalances as an explanation for the abnormal phenotype. [8,15,16]. Several mechanisms such as an intragenic disruption, a disruption of regulatory elements, fusion genes, or a position effect with variable expression of the gene near the translocation can be the underlying cause of abnormal phenotypes [15]. 

Optical genome mapping is able to detect all classes of structural variants (SVs) at a higher resolution than conventional cytogenetic methods do. Several studies showed a high concordance with the current combinatorial cytogenetic methods when looking at complex genetic disorders, constitutional disorders, and tumors. In addition, OGM is able to detect most of the different types of chromosomal anomalies including aneuploidies, large deletions/duplications, CNVs, balanced chromosomal events, and complex chromosomal rearrangements. It enables a more accurate determination of structural rearrangements such as translocation breakpoints [17]. Thus, it can also be recognized as another key method in genomic technology for the detection of reciprocal balanced translocations in patients with monogenic disorders such as MFS as well.

We report on a mother and daughter with clinical features of connective tissue disorder specifically from the Marfan syndrome spectrum. To the best of our knowledge, it is the first description of a disruption in chromosome region 15q21.1 of the *FBN1* gene by a translocation breakpoint.

With the help of OGM, we were able to confirm the breakpoints located between g.139888511 and g.139898832 of chromosome 2, as well as between g.48716726 and g.48724425 of chromosome 15 (hg19). Further sequencing results showed the breakpoint, within intron 55 of *FBN1,* and suggests a minor loss of approximately 2.2 kb on chromosome 15. Due to the imbalance on chromosome 15, the most probable explanation for the heterozygous loss of function of the *FBN1* gene on one allele would be the introduction of a premature stop codon through the fusion sequence resulting either in a truncated protein or in nonsense-mediated decay. Nevertheless, the clinical variability of MFS could be related to specific *FBN1* isoforms [18]. Due to the disruption of *FBN1*, an expression profile of *FBN1* isoforms in skin and adventitial fibroblasts of the patients would further elucidate the effect of the translocation.

The unambiguous, genetically supported diagnosis opens the way to close-meshed screening for the affected family members. Besides a direct phenotypic effect in the daughter, the reciprocally balanced translocation also has a critical impact on the daughter’s reproduction. In the case of a balanced transmission of the reciprocal translocation to offspring, a similar phenotype of connective tissue disease is to be expected. In the case of an unbalanced transmission to an offspring, a severe chromosome abnormality with possible miscarriage would be the most probable outcome. Continuation of a pregnancy with an offspring with multiple congenital malformations is very unlikely due to the expected huge imbalances. Therefore, the possibility of prenatal diagnostics or preimplantation genetic diagnosis (PGD) should be discussed with the daughter before family planning.

In the future, this fine-structural examination of the chromosomes could also help other families with a clinical suspicion of Marfan syndrome and inconspicuous molecular genetic results. This could be of crucial importance for the future diagnosis and therapy of Marfan syndrome. Currently, the precise characterization of ABCR breakpoints with OGM is still time-consuming and rarely proposed in daily diagnostic settings since it is not yet available in all laboratories. However, rapid technical development will make implementation in daily diagnostics possible soon.

## 5. Conclusions

We emphasize the importance of a combination of conventional cytogenetics and molecular cytogenomic methods (e.g., OGM) to reveal the cause of monogenic diseases typically caused by single nucleotide variants.

The combination of those different techniques in cases of reciprocally balanced translocations involving the *FBN1* gene is helpful to understand the extent of the molecular etiology of Marfan syndrome. Thus, it has the potential to identify novel, clinically relevant *FBN1* gene abnormalities that expand the diagnostic possibilities in patients with Marfan syndrome.

## Data Availability

The data presented in this study are available on request from the corresponding author.

## Supplementary Materials

The following are available online at https://www.mdpi.com/article/10.3390/genes12111836/s1, Figure S1: Phenotype of the mother and the daughter; Figure S2: Family pedigree.
