# Supplementary figures and images for: Marfan Syndrome Caused by Disruption of the FBN1 Gene due to A Reciprocal Chromosome Translocation
Source: Genes (Basel). 2021 Nov 21;12(11):1836. doi: 10.3390/genes12111836 (PMC8618173; doi:10.3390/genes12111836)

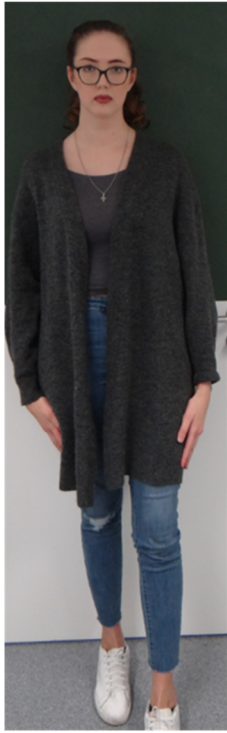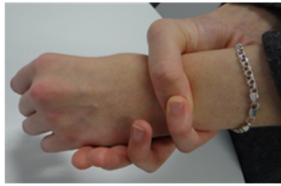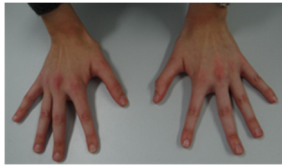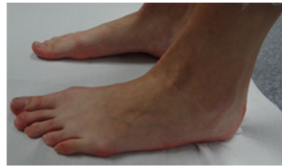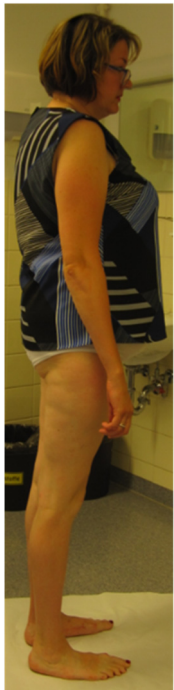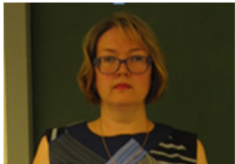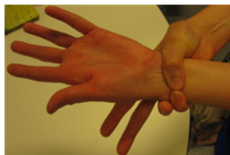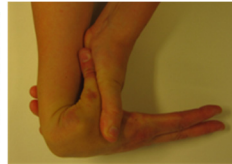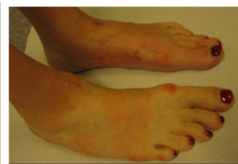

Supplement: Supplementary file 1 [file genes-12-01836-s001.zip › figure_S1.pdf]

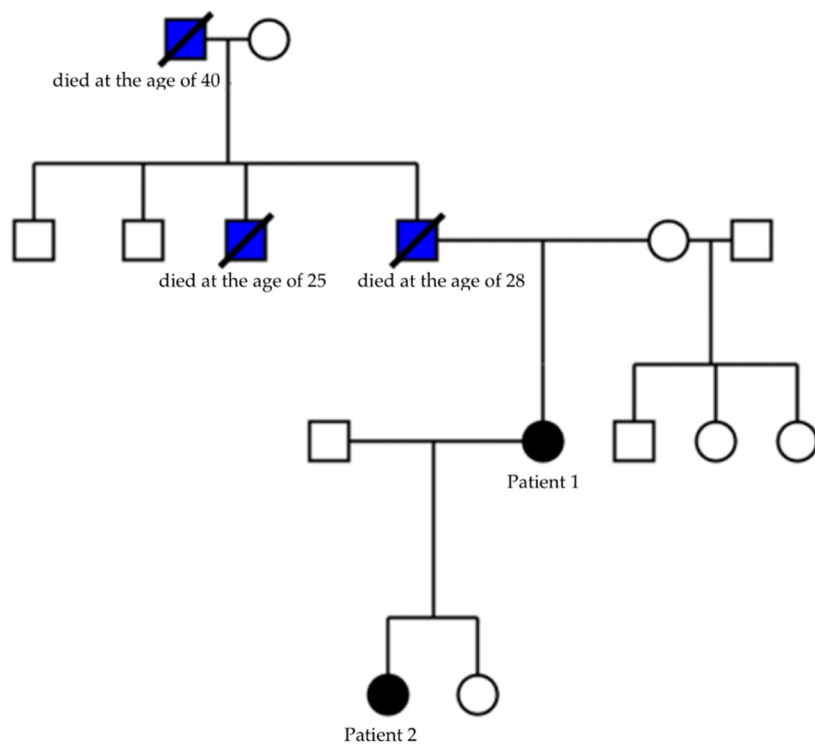

Supplement: Supplementary file 1 [file genes-12-01836-s001.zip › figure_S2.pdf]
